# Randomized Controlled Trial of Robot-Assisted Gait Training versus Therapist-Assisted Treadmill Gait Training as Add-on Therapy in Early Subacute Stroke Patients: The GAITFAST Study Protocol

**DOI:** 10.3390/brainsci12121661

**Published:** 2022-12-03

**Authors:** Barbora Kolářová, Daniel Šaňák, Petr Hluštík, Petr Kolář

**Affiliations:** 1 Department of Rehabilitation, University Hospital Olomouc, I.P. Pavlova 6, 779 00 Olomouc, Czech Republic; 2 Department of Neurology, Faculty of Medicine and Dentistry, Palacký University Olomouc and University Hospital Olomouc, 779 00 Olomouc, Czech Republic; 3 Comprehensive Stroke Centre, Department of Neurology, University Hospital Olomouc, I.P. Pavlova 6, 779 00 Olomouc, Czech Republic

**Keywords:** neurorehabilitation, stroke, gait recovery, robot-assisted gait training, magnetic resonance imaging

## Abstract

The GAITFAST study (gait recovery in patients after acute ischemic stroke) aims to compare the effects of treadmill-based robot-assisted gait training (RTGT) and therapist-assisted treadmill gait training (TTGT) added to conventional physical therapy in first-ever ischemic stroke patients. GAITFAST (Clinicaltrials.gov identifier: NCT04824482) was designed as a single-blind single-center prospective randomized clinical trial with two parallel groups and a primary endpoint of gait speed recovery up to 6 months after ischemic stroke. A total of 120 eligible and enrolled participants will be randomly allocated (1:1) in TTGT or RTGT. All enrolled patients will undergo a 2-week intensive inpatient rehabilitation including TTGT or RTGT followed by four clinical assessments (at the beginning of inpatient rehabilitation 8–15 days after stroke onset, after 2 weeks, and 3 and 6 months after the first assessment). Every clinical assessment will include the assessment of gait speed and walking dependency, fMRI activation measures, neurological and sensorimotor impairments, and gait biomechanics. In a random selection (1:2) of the 120 enrolled patients, multimodal magnetic resonance imaging (MRI) data will be acquired and analyzed. This study will provide insight into the mechanisms behind poststroke gait behavioral changes resulting from intensive rehabilitation including assisted gait training (RTGT or TTGT) in early subacute IS patients.

## 1. Introduction

After ischemic stroke (IS), the majority of people have some walking disabilities. Approximately 30% of IS patients are unable to walk without assistance six months after a stroke [1], and more than 60% of individuals who achieve independent ambulation after IS still walk at speeds insufficient to function effectively in the community [2]. Walking is one of the most important activities of daily living (ADL), and restoring gait function has become a critical goal of poststroke rehabilitation [2,3].

Early neurorehabilitation interventions seem to be critical to enhance neural plasticity and brain repair processes and to potentiate physiological motor behavioral restitution (i.e., restoring the physiological movement pattern) rather than mere compensation (adaptive mechanisms to utilize the residual capacity to accomplish a motor task) [3,4]. Functional brain imaging provides the possibility to describe neuroplastic brain adaptations related to motor recovery [5] and rehabilitation [6].

Current neurorehabilitation approaches mostly focus on task-specific training and intensive and repetitive exercise [7,8,9,10,11] with increases in both the therapy dosage and intensity [3,11]. Additional (add-on) physical rehabilitation, as compared to usual care or a control intervention, demonstrated the potential to further improve motor function, standing balance, or walking speed [11]. As the vast majority of poststroke patients experience some recovery, mostly occurring in the first weeks after stroke [4,12,13], the optimal time window to begin rehabilitation seems to be the early subacute stage (within the first weeks after stroke onset [14]).

Gait training is fundamental to promote poststroke gait recovery. The character of gait training should always reflect the individual patient’s walking ability [15] and might be realized with the assistance of a therapist or a robot, on the ground or on the treadmill, and with or without body weight support [10]. Conventional gait training also includes therapist-assisted gait training on a treadmill (TTGT) [16], which represents a repetitive task-specific activity with the possibility of a high number of repetitions produced through taken steps [17]. TTGT provides the opportunity to optimize the walking pattern with appropriate therapeutic handling. Treadmill gait training, even without a therapist’s assistance, has mostly been shown to improve walking abilities in subjects after stroke in the chronic phase [17,18,19]. Using treadmill gait training allows increases in the intensity of practice if overground walking is limited [19]. As the intensive treadmill gait training is stopped in chronic stroke survivors, the effect of this training declines [17]. It remains unclear whether intensive task-specific training has the potential to facilitate motor recovery through the restoration of physiological movement patterns or if it just improves physical fitness with a persisting compensatory walking pattern in chronic stroke patients.

Currently, robot-assisted gait training represents a rapidly advancing approach in neurorehabilitation [15]. Many patients early after stroke have a decreased ability to correctly produce volitional movements, and robot- or therapist-assisted gait training reduces unwanted compensations. Gait training should be, especially in the early subacute stage, continuous and repetitive. During robot-assisted gait training, an exoskeleton enables the application of a guidance force provided by the robotic orthosis to induce walking-like motion. Robot-assisted treadmill gait training (RTGT) systems are commonly used in combination with body weight support [15], allowing patients to engage in the repeated practice of complex gait patterns at near-normal speed over a longer period with less energy consumption and reduced effort given by the therapist [14,16]. Compared to treadmill gait training, RTGT may provide a longer training duration, the production of a greater number of step repetitions, more reproducible symmetrical gait patterns, and a reduction in the energy expenditure imposed upon the patient [15,16,20]. Some recent studies advocated for the use of RTGT versus treadmill or conventional gait training [21,22] and reported that robot-assisted gait training with concomitant physical therapy is more effective for gait recovery in stroke patients than physiotherapy alone [11,14,16,22,23,24]. Furthermore, Kim et al. [10] stated that RTGT, in comparison to bodyweight-supported treadmill training in hemiparetic poststroke participants, leads not just to improved functional clinical outcomes but also to increased cortical activity in the affected hemisphere, which may suggest that RTGT potentiates locomotor-task-related neuroplasticity [9,11].

To our knowledge, to date there is no study that has compared these two types of assisted gait training. Since they both facilitate a normal gait pattern, both are used in the clinical practice, and each possesses different strengths and limitations. Furthermore, limited evidence of the factors predicting the success of gait recovery after stroke exists, and a better understanding of the process of gait recovery early after IS in the context of intensive assisted gait rehabilitation remains the key research objective. The protocol of our study presents the first head-to-head comparison of RTGT versus TTGT, both added to conventional rehabilitation in the early subacute stage, evaluating their effects on the course of gait recovery for up to 6 months after a first-ever ischemic stroke. Our study reflects the current need for robust randomized clinical trials with clearly defined dosages and characters of rehabilitative interventions that are reliably evaluated using relevant clinical data, biomechanical parameters, and functional brain imaging data for further understanding the mechanisms behind poststroke gait behavioral changes and to define possible predictors of gait recovery, i.e., to identify brain structures with possible impacts on gait recovery using functional magnetic resonance imaging (fMRI), diffusion-weighted imaging (DWI), and surface-based morphometry up to 6 months after a first-ever ischemic stroke. The results of the proposed study will support decision making in poststroke rehabilitation regarding the timing, type, and setting of the interventions that are provided and facilitate realistic goal setting.

## 2. Materials and Methods

### 2.1. Study Design

The GAITFAST study (gait recovery in patients after acute ischemic stroke) was designed as an investigator-initiated, academic, single-blind, single-center, prospective, randomized clinical trial with two parallel groups and a primary endpoint of gait speed recovery up to 6 months after ischemic stroke. The protocol of the proposed GAITFAST trial is registered on ClinicalTrials.gov with identifier NCT04824482. The trial design follows the Recommendations for Interventional Trials (SPIRIT), the TIDieR checklist was used in conjunction with the SPIRIT. This study will be performed at the University Hospital Olomouc, Czech Republic. All participants will be enrolled at the Comprehensive Stroke Centre, and the neurorehabilitation interventions, including assisted gait training (RTGT or TTGT), will be performed at the Department of Rehabilitation.

### 2.2. Selection of Subjects

#### 2.2.1. Participants and Eligibility Criteria

The study population will consist of patients after first-ever IS who will be enrolled within 5–10 days after stroke onset at the Comprehensive Stroke Centre at the University Hospital Olomouc according to the following inclusion criteria: (1) first episode of unilateral ischemic stroke is detected on magnetic resonance imaging (MRI), resulting in walking deficits; (2) NIHSS score of 1–12 points at the time of enrolment; (3) lower limb movement impairment, with a score of at least 1 point on the NIH Stroke Scale (NIHSS) at the time of enrolment; (4) dependency in walking according to the Functional Ambulatory Category (FAC), with a score in the interval <1,3> (supervision or assistance, or both, must be given when walking) at the time of enrolment; and (5) age between 18 and 80 years. The exclusion criteria will include: (1) a previous history of any stroke, either ischemic or hemorrhagic; (2) other diseases modifying or limiting walking ability, currently receiving rehabilitation, or participation in another study; (3) significant or symptomatic ischemic heart disease; (4) significant or symptomatic peripheral arterial disease; (5) obesity (BMI ≥ 40) or weight higher than 135 kg (the weight limit for the robot-assisted gait training); (6) sensory aphasia with the inability to understand (confirmed by a certified speech therapist); (7) moderate or severe depression present at the time of enrolment assessed using the Beck scale, with a score above 10; (8) known cognitive impairment; (9) previous disability or dependence in daily activities assessed using the modified Rankin Scale, with a score of ≥3; (10) currently receiving dialysis; (11) diagnosed and/or receiving treatment for cancer; and (12) the presence of other orthopedic or neurological conditions affecting the lower extremities. Standard exclusion criteria for MRI examination will also apply.

#### 2.2.2. Screening and Recruitment

The screening of eligible participants with acute IS and their randomization for the GAITFAST trial will be performed by a certified neurologist at the Comprehensive Stroke Centre (University Hospital Olomouc). The neurologist will provide an overview of the study and determine initial interest in participation. All the potential risks and benefits related to study participation will be discussed with the participants. Potential participants will be informed that participating in the study is completely voluntary and that they may discontinue participation at any time. For individuals who express interest, the neurologist will determine initial eligibility and answer any participant questions. For individuals who agree to participate in this study and provide consent, the neurologist will perform screening procedures to confirm that the patient meets the eligibility criteria. By signing the informed consent, the participants will provide permission for the study investigators to obtain, collect, and analyze relevant data and to share them anonymously with the other members of the research team as well as to present or publish them anonymously at scientific conferences or in scientific journals. Regular screening of the study eligibility of all admitted patients with IS at the Comprehensive Stroke Centre by neurologists and physiotherapists (study investigators) will be performed every 2–3 days. Enough time will be given for the discussion with each eligible patient on his/her participation in the study, the study flow-chart, the importance of study follow-up, and informed consent.

### 2.3. Interventional Methods

#### Interventions

Enrolled participants will be randomly allocated into two groups before the start of the intensive rehabilitation at the Department of Rehabilitation at the University Hospital Olomouc, Czech Republic

Robot-assisted treadmill gait training (RTGT) is locomotor training guided by a robotic device Lokomat (Hocoma, Volketswil, Switzerland) on a treadmill according to a preprogrammed gait pattern with a robot-driven exoskeleton orthosis. The process of gait training is automated and controlled by a computer. The parameters of RTGT, such as guidance force or gait speed, are adaptively adjusted with respect to the patient’s actual walking abilities by a trained physiotherapist with at least 2 years of clinical experience on the Lokomat. Moreover, the dynamic body weight support system within this type of therapy is adjustable to the actual patient’s body-weight-bearing tolerance. RTGT is always realized under the supervision of a physiotherapist.

Therapist-assisted treadmill gait training (TTGT) is locomotor training via the repetitive execution of walking movements that are manually guided by a physiotherapist during treadmill gait training. Body weight support might be provided to patients during this type of gait training, if needed. Body weight support and gait speed are also adaptively adjusted with respect to the patient’s actual walking abilities as is the assistance, which is manually provided by physiotherapists with at least 4 years of clinical neurorehabilitation practice with poststroke patients. TTGT still represents a comparable type of training to RTGT, where each step might be adaptively corrected by the physiotherapist. However, the symmetrical stereotypical gait pattern might only be achieved in RTGT.

Assisted gait training (RTGT or TTGT) is performed five times per week, with durations of up to 30 min [23] for two weeks as an add-on therapy to conventional physical therapy. Adherence to the therapy is ensured by the adjustment of RTGT or TTGT according to the patient’s comfortable speed, actual need of body weight support, and his or her training intensity tolerance.

Participants in both groups will follow a two-week intensive conventional rehabilitation program with an add-on intervention (RTGT or TTGT) at the Rehabilitation Department of University Hospital Olomouc. The intensive conventional inpatient rehabilitation program will include individual physical therapy, which is based on neurophysiological approaches to improve movement patterns and is realized two times per day, with each session lasting for up to 45 min every weekday, and occupational therapy focused on improving abilities in activities of daily living, lasting for up to 45 min per day. Speech therapy or psychotherapy will be included if needed.

### 2.4. Blinding Procedure

All enrolled participants will be assessed by investigators that will be blinded to the group allocation. The physiotherapists performing the interventions (RTGT or TTGT) will not be blinded; however, they will not participate in the assessments at all and will not have any access to the interim and final results and outcomes. Although it is impossible to blind the participants due to the interventional study design, the participants will be instructed not to disclose their allocation to the investigators or the outcome assessors.

### 2.5. Study Protocol

All enrolled participants will be examined by evaluators that will be blinded to the group allocation. The assessments will be realized by certified neurologists, rehabilitation physicians, physiotherapists, and biomedical engineers with respect to their specialization at four assessment timepoints. Clinical visits will occur as follows:

Visit 0 (T0): enrolment 5–10 days after onset of qualifying IS at the Comprehensive Stroke Centre.

Visit 1 (T1) (3–5 days after V0): assessment before the initiation of an intensive rehabilitation program including assisted gait training (RTGT or TTGT) at the Department of Rehabilitation.

Visit 2 (T2) (14 ± 7 days after visit 1 and 1 month after visit 0): assessment at the end of the intensive rehabilitation program including gait training at the Department of Rehabilitation.

Visit 3 (T3): assessment 12 ± 1 weeks after visit 0 (3 months after visit 0).

Visit 4 (T4): assessment 24 ± 2 weeks after visit 0 (6 months after visit 0).

Scheduled outpatient visits in the GAITFAST trial will be performed by a neurologist at the Department of Neurology (visit 1–visit 4), a rehabilitation physician, a physiotherapist, and a biomedical engineer at the Kinesiology Laboratory at the Department of Rehabilitation (visit 1–visit 4). Multimodal MRI data will be acquired on a 3T scanner (Siemens Magnetom Vida, Erlangen, Germany) at the Department of Radiology. Scanning will be supervised by a member of the Functional Magnetic Resonance Imaging Laboratory (visit 1–visit 4). The duration of patient participation will be 6 months (Table 1).

Each visit will include an assessment to evaluate gait speed, gait dependency, neurological impairment, sensorimotor function, and gait biomechanics and a multimodal MRI of the brain. The outcome variables and study flow are presented in more detail in Table 2.

As data accumulate, the validity and the integrity of the trial will be subjected to an interim analysis after the first 30 participants have been assessed at all four assessment timepoints. With respect to the results of the interim analysis, the study design (such as the sample size, allocation ratio, or eligibility criteria) might be adaptively modified.

Adverse events (e.g., falls, pain, or dizziness) that occur during the study period, whether related to the study intervention or not, will be recorded. In case the training intervention is discontinued for any reason (e.g., hospital discharge, medical complications, or serious adverse event), a participant will not be required to participate in the follow-up assessments. Patients who suffer from a recurrent stroke during the study will be excluded, as will those with any medical condition or disease that occurs during the study and may affect limb movement or mobility. All enrolled patients may undergo outpatient rehabilitation after discharge from the Department of Rehabilitation. The types of rehabilitation treatment during follow-up will be recorded.

### 2.6. Study Procedure Outcome Measures

The outcome variables and their study assessments are listed in Table 2.

#### 2.6.1. Primary Outcome

Walking speed is defined as a primary outcome in this study and is the most used outcome measure of walking ability in locomotor rehabilitation. Walking speed has been shown to be a predictor of independence, the functional level at home and within the community, and quality of life [25]. Gait speed will be evaluated before the beginning of inpatient RHB, after two weeks (at the end of inpatient RHB), and during follow-up (three and six months after stroke onset). Gait speed will be measured using the 10-meter walking test. The subject will be asked to walk a distance of 10 m at his/her comfortable speed. The time will be measured for the distance of the middle six meters, which will reflect walking acceleration and deceleration. If the physical assistance of another person (to prevent a fall or collapsing) is needed for a patient to complete the test, the level of assistance will be documented. If total assistance or assistance for limb swing or forward propulsion is required, a walking speed of 0 m/s will be documented [26]. The usage of any assistive device and/or bracing (that patients use for walking and are needed to complete the test) will also be documented. Based on the gait speed, walking may be classified as household (<0.4 m.s^−1^), limited community (0.4 to 0.8 m.s^−1^), or full community ambulation (>0.8 m.s^−1^) [20].

#### 2.6.2. Secondary Outcomes

- Walking dependency

Walking dependency will be evaluated by the functional ambulation categories (FAC) using a 6-point scale ranging from 0 to 5. Scores in the interval <1,3> indicate a dependent ambulator who requires assistance from another person during walking (score 1—continuous manual contact, score 2—intermittent manual contact, and score 3—verbal supervision/guarding). Scores of 4 or 5 indicate an independent ambulator (score 4—independent walking on a level surface and score 5—independent walking on any surface). The FAC has been proven to have excellent reliability, good concurrent and predictive validity, and even good responsiveness in poststroke patients with hemiparesis [27]. The FAC scores have great potential to change significantly after the first 2 weeks of the early inpatient rehabilitation program [27].

- Brain activity related to gait recovery

Specific brain structures with possible impacts on gait recovery in IS patients will be identified using functional magnetic resonance imaging (fMRI) during simple lower limb movements, gait imagery, and gait observation as well as using diffusion-weighted imaging (DWI) and surface-based morphometry. The functional MRI activation magnitude, calculated as the difference in the BOLD signal between task and rest, will be assessed within predefined gait-related brain regions of interest (ROIs), i.e., the sensorimotor cortex, premotor cortex, supplementary motor area, brainstem, and cerebellum. Changes in these ROI parameters over time will be statistically tested within the group, and the regional post-training minus pretraining difference in each group will be submitted to a between-group analysis. Gait recovery after treadmill exercise has been associated with increased task-related activation in the posterior cerebellum and brainstem [28] as well as in the primary sensorimotor cortices and basal ganglia [29]. Moreover, the task-related activation volume in the ipsilesional primary sensorimotor foot representation has been identified as an independent baseline predictor of gait recovery [30]. However, simple leg movements may not be sufficiently representative of a more complex motor behavior such as gait. As it is impossible to directly measure gait-related brain activation in fMRI, gait imagery has been employed as a substitute for real movements in healthy subjects [31] as well as stroke patients with gait impairment [32]. Beyond fMRI, other potential imaging biomarkers include white matter integrity, assessed using diffusion-weighted imaging (DWI), and surface-based morphometry based on structural imaging [33]. So far, according to systematic reviews, evidence for biomarkers based on fMRI, DWI, and structural MRI data remains inconclusive, mostly due to methodological limitations [16].

- Neurological impairment

The National Institute of Health Stroke Scale (NIHSS) represents the most widely used rating scale to evaluate neurological impairment after IS [34]. The NIHSS includes items on consciousness, language, movement, sensation, ataxia, eye movement, the visual field, and others. The scores range from 0 to 42, with higher scores indicating more severe neurological deficits.

- Sensorimotor impairment

The Fugl-Meyer Assessment of Lower Extremity (FMA-LE) is used worldwide and represents the gold standard for the evaluation of poststroke motor impairment [3], both for clinical use and research. The FMA-LE showed excellent intra- and inter-rater reliability in patients early after stroke in an inpatient rehabilitation setting [35]. The FMA-LE consists of five domains (motor function, sensation, balance, joint range of motion, and joint pain) divided into 17 items, with a maximum score of 34 points [36]. Each item is scored on a 3-point scale (0—cannot perform, 1—can partially perform, and 2—can fully perform).

- Biomechanical gait parameters

Biomechanical kinematic and kinetic gait parameters will be evaluated using the instrumented treadmill gait analysis system Zebris FDM-T (Zebris Medical GmbH, Isny, Germany). It has previously been suggested that one of the key questions arising from gait recovery issues is to what extent is the improvement in gait speed the result of behavioral restitution (i.e., motor control improvement in paretic limbs) or the result of more or less fixed compensatory mechanisms (with an asymmetrical gait pattern) [3]. Measuring the biomechanical variables within this study should address this issue. Spatiotemporal gait parameters such as gait speed, cadence, the stance phase duration for the hemiparetic and nonparetic limbs, the step length for hemiparetic and nonparetic limb, and the double stance phase will be expressed as percentages of the gait cycle. Kinetic variables such as the ground reaction force (N) or plantar pressure distribution (N/cm^2^) for both the paretic and nonparetic limbs will also be analyzed. All these parameters will be measured at patients’ preferred speed.

#### 2.6.3. Other Outcomes

- Cognition

Changes in the Montreal Cognitive Assessment (MoCA), which is a cognitive screening test designed to detection cognitive impairment will be recorded. The MoCA has a good predictive value for the development of poststroke cognitive impairment in the follow-up when used in the acute stage after stroke [37]. It assesses different cognitive domains: attention and concentration, executive functions, memory, language, visuo-constructional skills, conceptual thinking, calculations, and orientation.

- Dependency in daily living activities

The Modified Rankin Scale is used for measuring the degree of disability or dependence in the daily activities of patients after stroke. It is the most widely used clinical outcome measure after stroke. The scale has six points, and a higher score indicates a worse outcome. The minimum score is 0 points, indicating no symptoms at all, and the maximum score is 6 points, indicating death [38]. The Barthel index is a scale used to measure performance in activities of daily living (ADL). The maximum score is 100 points. The BI has excellent inter-rater reliability as a stroke outcome measure [38].

- Clinical assessment of gait-related task

The Timed Up and Go (TUG) test is a clinical test that is used to assess a person’s mobility, which requires both static and dynamic balance. The objective of this test is to determine fall risk and measure the progress of balance, sit to stand, and walking. There is no defined value of the minimal clinically important difference for stroke patients; however, [39] showed that a change of ≥28% can indicate a relevant difference.

- Lower limb muscle strength

Lower limb muscle strength testing, where a grading of 0–5 reflects the maximum force executed by a certain muscle [40,41], will be conducted. Lower limb muscle strength is associated with gait recovery, and a grade ≥3 represents a predictor of independent walking after stroke [40].

- Lower limb muscle activity during walking

Changes in lower limb muscle activity (medial gastrocnemius, tibialis anterior, quadriceps, and hamstrings) during treadmill gait and overground walking will be measured by bipolar surface electromyography (Delsys Trigno Avanti wireless EMG/IMU sensor system, Delsys, Natick, MA, United States) according to International Society of Electrophysiology and Kinesiology [42]. Inertial measurement units (IMUs) have been validated to measure movement quality in clinical settings [43]. EMG/IMU sensors with built-in triaxial accelerometers and triaxial gyroscopes will also be used to identify the course of the stance and the swing phase during treadmill and overground walking for both the paretic and nonparetic limbs [44]. The changes in the lower limb muscular activation patterns resulting from robotic or conventional gait training may help to identify the physiological gait characteristics in patients after stroke [43,45].

- Depression

Changes in score on the Beck Depression Inventory Scale—a 21-item self-report rating inventory that measures characteristic attitudes and symptoms of depression—will be recorded. Each item (question) has a set of at least four possible responses that range in intensity. Scores of 0–9 indicate minimal depression, and scores of 30–63 points indicate severe depression. Higher total scores indicate more severe depressive symptoms.

- Quality of Life

Changes in the score on the EQ-5D-3L Questionnaire’s standard layout for recording an adult person’s current self-reported health state will be recorded. This questionnaire consists of a standard format for respondents to record their health state according to the EQ-5D-3L descriptive system and the EQ VAS.

### 2.7. Sample Size

Based on an analysis of our pilot data concerning the gait velocity (primary outcome), 120 participants (60 in each group) are anticipated to be enrolled in this study. This number of participants would provide 85% power (α level 5%) to detect a difference of 0.16 m/s in the 10 MWT (with respect to a missing data rate of 15% and an expected 10% drop in the follow-ups) between groups after intensive rehabilitation with add-on intervention. The gait speed difference of over 0.16 m/s was previously defined as the minimal clinically important difference in gait speed in subacute stroke patients [46].

For the multimodal magnetic resonance imaging (MRI), including functional MRI (fMRI), we have estimated that a sample size of 50 participants (25 in each group) will suffice to find significant correlations of MRI outcomes with gait recovery. This is based on previous studies of fMRI and gait recovery in similar clinical cohorts [29,32]. We will perform multimodal MRI in half of the randomly selected enrolled IS patients (1:2 randomization), i.e., in 60 participants, to allow for a common drop-out of examined patients with MR images of poor or insufficient quality. As we do not have sufficient information to estimate the variance in the outcomes, we plan to calculate the definitive effect sizes based on the interim analysis of the first 30 subjects measured within all four visits (visit 1–visit 4).

### 2.8. Randomization

The randomization of the enrolled participants for robot-assisted treadmill gait training (RTGT) or therapist-assisted treadmill gait training (TTGT) will be performed as a block randomization with a 1:1 allocation to either RRGT or TTGT. As mentioned above, in randomly selected IS patients with a 1:2 allocation, a repeated multimodal magnetic resonance imaging (MRI) examination, including functional MRI (fMRI), will be performed during the study follow-up.

Participants will be randomized after the screening of eligible participants by the certificated neurologist at the Comprehensive Stroke Centre and before the first clinical visit (visit 1).

Participants will be randomized in a 1:1 ratio and in a 1:2 ratio to multimodal magnetic resonance imaging (MRI) using the web randomization module. A randomized web number generator will be implemented for group allocation. Neurologists will generate the allocation sequences, enroll the participants, and assign the participants to interventions.

The allocation might be adaptively modified in cases when patients randomized for RTGT, after enrolment and during the course of rehabilitation, will be identified as independent walkers (FAC > 3). This change will be reported. It has previously been identified that stroke survivors with gait deficits (FAC ≤ 3) with higher motor impairment are those who benefit the most from robot-assisted therapy in combination with conventional therapy [15,23,47,48].

All participating patients will obtain a card before discharge (visit 2) with the dates of all scheduled outpatient events (visit 3 and visit 4), including the dates of the fMRI examinations and even the telephone contacts with the study investigators (neurologist and principal physiotherapist). All enrolled patients will be promoted and motivated to remain in the study and complete follow-ups during each scheduled visit. In participants who discontinue or deviate from the intervention protocols, the acquired outcome data will be collected for a further subanalysis.

### 2.9. Data Management

The study protocol, data collection forms, and even files with outcome data will be stored on a locked disc shared with study investigators, and every 3 months all data will be backed up on external discs. All collected patient data will be shared and analyzed anonymously. All questionnaires and clinical scales of all participants will be stored for further analysis. All patient data will be scrambled to ensure confidentiality.

Personal information about potential and enrolled participants will be collected by only one investigator without further sharing within the study team. Patients´ personal information will be confidential to all other study investigators during the study duration as well as after the end of the trial. All collected patient data will be shared and analyzed strictly anonymously.

All reported adverse events or other unintended effects of the trial interventions will be recorded. Missing data for each variable will be reported.

There are no plans to give access to the full protocol, the participant-level data, or the statistical code.

### 2.10. Statistical Analysis

For the statistical analysis of all tested clinical parameters, gait movement pattern parameters, and extracted imaging parameters, the distribution of the data will first be explored. If normally distributed, a two-factor mixed-model ANOVA will explore the differences between the groups and time points. If not normally distributed, Friedman tests will be used to explore the differences between time points (visits 1–4) within each group and Mann–Whitney U tests will be used to explore the differences between both groups (RTGT and TTGT).

The statistical analysis of fMRI data will include standard preprocessing with subsequent general linear model fitting and a group-wise analysis in the Montreal Neurological Institute (MNI) 152 standard space after the nonlinear normalization of the individual structural MRI data [49]. Significant group effects will be evaluated using an analysis of covariance (ANCOVA) with group, visit, and age as covariates. An atlas-based region of interest (ROI) analysis will be used to evaluate the amplitude and volume of the primary sensorimotor and premotor activations and the laterality index (ipsilesional − contralesional/ipsilesional + contralesional), which are the main secondary outcome measures of the fMRI analysis. Since the functional study design and paradigm partly follow the study of Boyne et al. [32], the choice of regressors and contrasts for the statistical analysis of the corresponding data will likewise follow that study. Structural data (DWI and T1-weighted) will be evaluated in separate pipelines to extract the following parameters from anatomically defined ROIs: regional cortical thickness; fractional anisotropy (FA); and axial, radial, and mean diffusivity (AD, RD, and MD), respectively. Alternative DWI analysis models may include, e.g., FD/FDC or NODDI. The quantitative parameters derived from functional and structural imaging will be included in an extended factor analysis of the global predictors of gait recovery.

With respect to the type of missing variable, the appropriate imputation (e.g., single imputation, multiple imputation, etc.) might be performed if needed [50].

As data accumulate, the validity and the integrity of the trial will be subjected to an interim analysis after the first 30 participants have been assessed at all four timepoints. The interim analysis, including the first 30 completely measured participants, will guide the final statistical methods. An interim analysis based on 30 participants with complete data will be conducted to perform a power analysis and to compute the final sample size. The principal investigator will have access to the interim results. Additional analyses and further subgroupings are not planned.

## 3. Discussion

The early subacute or subacute period offers an optimal therapeutic time window for achieving the maximal effect of physical rehabilitation to promote motor recovery. Especially during the early subacute stages after IS, repetitive, high-dosed, task-specific training enhances neuroplasticity and may accelerate gait recovery after IS [12,13,14]. It was previously proven that intensive RTGT starting in the acute stage (<1 week after stroke onset) improves, in addition to other functions, sensorimotor function more effectively than in the subacute or chronic stages after stroke [51]. Moreover, if greater improvements in motion execution are made early during the subacute stage, better outcomes may be expected during the chronic stages of recovery [12,13,51,52,53]. The training intensity may be effectively increased by robot-assisted gait training in stroke survivors classified as dependent walkers with higher motor impairment [17,22,24,47,48]. Robot-assisted gait training with concomitant physiotherapy has a greater potential to recover independent walking in stroke patients than physiotherapy alone [17]. 

However, the evidence concerning the effect of RTGT in comparison to TTGT in dependent walkers is unsatisfactory. In RTGT, the gait pattern is realized with robotic exoskeleton assistance, but in TTGT the compensatory behavior is minimized by the therapist’s handling. The questions arising from the current evidence and even from clinical practice is to what extent might assisted gait training (RTGT or TTGT) with concomitant physiotherapy promote physiological gait pattern recovery early after stroke and if RTGT is superior to TTGT.

The neural correlates and mechanisms of successful motor recovery and rehabilitation after stroke have been studied noninvasively for several decades, most commonly with structural and functional MRI [5,6]. FMRI has become a common outcome measure in clinical trials [53]. The successful recovery of walking in chronic stroke patients is associated with increased cortical and subcortical sensorimotor activation during simple movements as well as during gait imagery [28,29,30,32]. We intend to extend these observations to subacute stroke patients. Besides functional MRI, the effect of lesion location on gait improvement after rehabilitation (see, e.g., Jones [54]) will be assessed using early regional cortical atrophy calculated from structural MRI and the decrease in white matter integrity (assessed by diffusion-weighted MRI).

An understanding of the patterns of recovery over time is a key issue within clinical practice. In the context of stroke rehabilitation, this information facilitates realistic goal setting and can guide decision making about the timing, type, and setting of the provided interventions.

### 3.1. Limitations

We consider the single-center study design as a potential limitation of our study. Some of the inclusion/exclusion criteria for study enrolment would also limit the possibility to extrapolate our results to the general stroke population.

### 3.2. Strengths and Relevance

The proposed study reflects the current strong need for randomized trials including follow-up measurements with clearly defined dosages and characters of rehabilitative interventions and a sufficient number of participants [3]. The study outcomes are based on clinical data, biomechanical data, and brain MR imaging data that may help in further understanding the mechanisms underlying poststroke gait behavioral changes.

An important consideration in neurorehabilitation stroke research focused on functional recovery after stroke is the distinction between improvements in function that result from changes in underlying impairments such as behavioral restitution and those that reflect alternate compensatory mechanisms [3]. This randomized trial also focuses on this very topic; besides the clinical assessments, the protocol includes a biomechanical gait analysis and the identification of the brain structural and functional predictors of gait recovery.

The results of the GAITFAST trial will provide new evidence of the effectiveness and benefits of robot-assisted treadmill gait training (RTGT) and therapist-assisted treadmill gait training (TTGT) for gait recovery during intensive rehabilitation in the early subacute phase of IS. The results will also help in better understanding the process of gait recovery in IS patients and thus facilitate realistic goal setting and guide decision making about the timing, type, and setting of the individual rehabilitation interventions.

## Figures and Tables

**Table 1:** Schedule of enrolment, interventions, and assessments at each time point (T0–T4) according to Recommendations for Interventional Trials (SPIRIT).

	STUDY PERIOD										
	Enrolment	Allocation		Post-Allocation						Follow-Up	
		Visit 0		Visit 1				Visit 2		Visit 3	Visit 4
TIMEPOINT	0	T_0_		T_1_		Intervention		T_2_		T_3_	T_4_
		5–10 days after IS onset		3–5 days after T_0_		2 weeks		14 ± 7 days after T_1_		3 months ± 1 weeks after T_1_	6 months ± 2 weeks after T1
ENROLMENT											
Eligibility screen	X										
Informed consent	X										
Allocation		X									
INTERVENTIONS											
TTGT						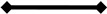					
RTGT						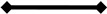					
ASSESSMENTS											
Outcome variables (listed in Table 2)				X				X		X	X

**Table 2:** Overview of outcome variables and outcome assessments throughout the study flow.

Data		Outcome Variables		T0	T1	T2	T3	T4
Demographic variables		age, gender, height, weight, time since stroke		x				
Stroke morphology/structure-related variables		site of stroke, location of lesion, size of lesion, time since stroke		x				
								
**Outcome domain**		**Outcome Assessments**	**Abbreviation**	**T0**	**T1**	**T2**	**T3**	**T4**
Primary Outcome								
	Gait velocity	10-meter Walking Test	10 MWT		x	x	x	x
Secondary outcomes								
	Walking dependency	Functional Ambulatory Category score	FAC		x	x	x	x
	Brain activity	Functional magnetic resonance imaging	fMRI					
	Neurological impairment	National Institute of Health Stroke Scale	NIHSS	x	x	x	x	x
	Sensorimotor function	Fugl-Meyer Assessment of Lower Extremity	FMA-LE		x	x	x	x
	Kinematic and kinetic gait parameters	Treadmill gait analysis system			x	x	x	x
Other outcomes								
	Cognition	MoCA test score	MoCA		x	x	x	x
	Dependency in daily living activities	Rankin scale, Barthel index	mRS, BI		x	x	x	x
	Clinical assessment of gait-related task	Timed Up and Go test	TUG		x	x	x	x
	Lower limb muscle strength	Muscle test			x	x	x	x
	Lower limb muscle activity	Surface electromyography	sEMG					
	Lower limb kinematics	Inertial measurement units			x	x	x	x
	Depression	Beck depression test			x	x	x	x
	Quality of life	EQ-5D-3L test			x	x	x	x

## Data Availability

The data generated during this study will be made available from the corresponding author on reasonable request.
